# Supplementary material for: Mutation Rate and Spectrum of the Silkworm in Normal and Temperature Stress Conditions
Source: Genes (Basel). 2023 Mar 4;14(3):649. doi: 10.3390/genes14030649 (PMC10048334; doi:10.3390/genes14030649)
Supplement: Supplementary file 1 [file genes-14-00649-s001.zip › Supplementary Tables S1-S6.pdf]

Supplementary table S1. Sequencing depth and coverage of all samples

Normal conditions				
**Samples**	**No.reads**	**Mapped reads**	**Mean coverage(X)**	**Mean Mapping Quality**
NC-Paternal	102,334,097	99.73%	32.9723	21.48
NC-Maternal	106,476,407	99.75%	34.2729	21.3
NC-offspring-1	113,240,333	99.81%	36.5216	21.52
NC-offspring-2	157,090,378	99.81%	50.654	21.49
NC-offspring-3	117,020,978	99.77%	37.6965	21.48
NC-offspring-4	121,410,039	99.81%	39.1693	21.57
NC-offspring-5	130,213,361	99.82%	41.9743	21.53
NC-offspring-6	135,843,883	99.80%	43.8225	21.57
NC-offspring-7	124,705,370	99.74%	40.1756	21.53
NC-offspring-8	97,341,821	99.82%	31.4037	21.58
NC-offspring-9	133,625,850	99.82%	43.0968	21.55
NC-offspring-10	102,744,542	99.73%	33.1089	21.58
NC-offspring-11	141,964,996	99.73%	45.7257	21.58
NC-offspring-12	118,854,098	99.79%	38.3124	21.52
NC-offspring-13	95,602,982	99.59%	30.6295	21.47
NC-offspring-14	138,901,831	99.82%	44.809	21.54
NC-offspring-15	115,422,424	99.77%	37.1748	21.52
NC-offspring-16	121,143,511	99.74%	38.9763	21.3
NC-offspring-17	110,462,424	99.79%	35.5785	21.3
NC-offspring-18	129,239,764	99.77%	41.6265	21.33
NC-offspring-19	130,208,024	99.73%	41.9198	21.31
NC-offspring-20	118,664,268	99.75%	38.1988	21.27
NC-offspring-21	134,638,604	99.51%	43.3233	21.25
NC-offspring-22	116,424,472	99.75%	37.5395	21.27
NC-offspring-23	143,429,685	99.76%	46.1688	21.25
NC-offspring-24	116,051,579	99.75%	37.368	21.25
NC-offspring-25	103,932,734	99.76%	33.5194	21.24
NC-offspring-26	128,354,485	99.73%	41.3713	21.26
NC-offspring-27	135,234,628	99.75%	43.5493	21.27
NC-offspring-28	136,149,869	99.74%	43.872	21.31
NC-offspring-29	113,064,167	99.78%	36.4006	21.26
NC-offspring-30	133,326,762	99.78%	42.9638	21.35

Low temperature				
**Samples**	**No.reads**	**Mapped reads**	**Mean coverage(X)**	**Mean Mapping Quality**
LT-Maternal	102,904,534	99.78%	33.2178	21.12
LT-Paternal	104,289,419	99.83%	33.7196	21.14
LT-offspring-1	83,989,840	99.81%	27.1266	21.14
LT-offspring-2	75,002,344	99.73%	24.1939	21.03
LT-offspring-3	83,596,507	99.76%	26.9958	20.72
LT-offspring-4	78,746,188	99.76%	25.4143	21.06
LT-offspring-5	92,966,203	99.84%	30.0389	20.94
LT-offspring-6	69,939,577	99.49%	22.5362	21.35
LT-offspring-7	87,804,757	99.86%	28.404	21.21
LT-offspring-8	78,887,093	99.86%	25.5083	21.32
LT-offspring-9	81,070,607	99.83%	26.2112	21.42
LT-offspring-10	73,645,332	99.80%	23.784	21.24

Supplementary table S1 continue

High temperature				
**Samples**	**No.reads**	**Mapped reads**	**Mean coverage(X)**	**Mean Mapping Quality**
HT-Maternal	94,687,253	99.79%	30.5639	21.02
HT-Paternal	108,112,620	99.73%	34.8955	21.27
HT-offspring-1	83,005,550	99.79%	26.8074	21
HT-offspring-2	84,024,521	99.82%	27.1427	20.84
HT-offspring-3	76,748,540	99.81%	24.7832	20.93
HT-offspring-4	77,410,675	99.84%	25.0004	21.03
HT-offspring-5	70,368,965	99.83%	22.737	20.8
HT-offspring-6	81,034,084	99.85%	26.183	21.28
HT-offspring-7	72,583,253	99.83%	23.443	21.24
HT-offspring-8	84,178,201	99.80%	27.1971	21.45
HT-offspring-9	85,732,489	99.84%	27.7105	21.26
HT-offspring-10	82,210,107	99.81%	26.5712	21.21

Supplementary table S2. The information of all *de novo* mutations.

Normal conditions															
**Variant Numbe**	**Samples**	**Chromesome**	**Position**	**Ref**	**Alt**	**Read depth**	**Ref reads**	**Alt reads**	**Proportion of Alt read**	**Region**	**TE**	**Validatio**	**Forward primer**	**Reverse primer**
NC1	NC-offspring-9	Bomo_Chr23	3722500	T	G	30	21	9	0.30	intergenic	OUT	F	CAGTTTTCCGGTTGGTCCCT	GCGATACAAAACCCGACAGC
NC2	NC-offspring-13	Bomo_Chr17	13472593	A	G	36	25	11	0.31	intergenic	OUT	T	CCGGTACTCGAGACAGCATT	GTGGTTGTACCTACCCGTCC
NC3	NC-offspring-13	Bomo_Chr21	14309584	G	A	26	18	8	0.31	intergenic	OUT	T	TCGTTGCGGGGTCTAAAGTC	CAACAGCATTCACGTTGCGA
NC4	NC-offspring-6	Bomo_Chr22	17131823	T	C	29	20	9	0.31	intergenic	OUT	T	GCCTCCGTCCTGAGAAGAAC	CTCCCCGATTCAGGATCACG
NC5	NC-offspring-20	Bomo_Chr28	10516179	A	T	32	22	10	0.31	intergenic	IN	T	CGTCCCATCCTCCTAATGCC	CTTTTTGGCGGGAACACGAG
NC6	NC-offspring-7	Bomo_Chr13	2692518	G	A	35	24	11	0.31	intergenic	OUT	T	CCACATCGGAGCTAACTGCT	AAGTGCGACACGTCTTTGGA
NC7	NC-offspring-9	Bomo_Chr11	14924852	T	A	38	26	12	0.32	intergenic	IN	T	GACGCTTTTTACCGGTGTGG	CGGCGAAATGTACCGAGCTA
NC8	NC-offspring-27	Bomo_Chr19	3738838	A	C	38	26	12	0.32	intergenic	IN	F	CAGCTTATCCTCGACTGCGT	AGCGGTTAGTTGCCCTCAAT
NC9	NC-offspring-13	Bomo_Chr4	17341932	A	G	25	17	8	0.32	intron	OUT	T	GGCTCGCTGACCAAAGTAGT	CATAGCCCGTTAGATGGCGT
NC10	NC-offspring-13	Bomo_Chr15	8809936	G	A	24	16	8	0.33	intergenic	OUT	T	AGTTACGGCAAAGTGTGGCT	ACAGTTGACAGATGACGGCTT
NC11	NC-offspring-24	Bomo_Chr13	12343252	A	T	33	22	11	0.33	intergenic	OUT	T	CGGGCAACTAACTGTCAGGA	TGGACGACGACTGCGTTTAT
NC12	NC-offspring-21	Bomo_Chr10	13566275	A	T	45	30	15	0.33	intergenic	OUT	T	GACTGTCCCGTTGTTTTGCC	CCTACGGTGTTTTGCCTCCT
NC13	NC-offspring-23	Bomo_Chr17	10023	A	G	39	26	13	0.33	intergenic	OUT	F	TCTCGCAGAGTAGAGCACCT	TTTCCACGTCCTCGATGGTG
NC14	NC-offspring-15	Bomo_Chr24	16497379	T	G	44	29	15	0.34	intron	IN	T	GCCAAGGGGGAGATTTGGAA	TCGTAGGTCTCGAAGGGGAG
NC15	NC-offspring-18	Bomo_Chr19	6823092	C	G	35	23	12	0.34	intergenic	OUT	T	TGTGTCTCGTGAACCGTCTT	TAGGTGCCTCGTTGGCATAG
NC16	NC-offspring-12	Bomo_Chr10	11685117	T	A	32	21	11	0.34	intergenic	OUT	T	GTCAAAGCGATCCGAGGCTA	TACATCGCCAACGGAACGAA
NC17	NC-offspring-24	Bomo_Chr5	4669486	G	A	26	17	9	0.35	intergenic	OUT	T	GCGAGGGTTGAACGAAACTG	TGAACGCGCTAATCTCAGGA
NC18	NC-offspring-16	Bomo_Scaf010	93298	A	G	28	18	10	0.36	intergenic	IN	T	CGGGGCTAAGCGACAATAAC	CGGCCTTACTTCTCAAAGGGT
NC19	NC-offspring-29	Bomo_Chr10	10772046	C	A	53	34	19	0.36	intron	OUT	T	TCTGTGGGGTCCAGTAACCA	AGTACCGGCGAAAGGAAGTC
NC20	NC-offspring-13	Bomo_Chr7	12904904	C	T	25	16	9	0.36	exon	OUT	T	TTGCCCTACTGGTGGTAGGA	GCAGCGATGTAACACGAAGG
NC21	NC-offspring-22	Bomo_Chr5	7531454	T	C	36	23	13	0.36	intron	OUT	T	TTGGGGCTCAACACTTTTCTCT	CGTACAGCGGCTTTGTTTCAT
NC22	NC-offspring-30	Bomo_Chr12	6965686	C	G	30	19	11	0.37	intron	OUT	T	AAGCGTACTGAAGGCCCAAA	GGCCTGGACACGCTTTAGTA
NC23	NC-offspring-28	Bomo_Chr7	1064237	C	A	43	27	16	0.37	exon	OUT	T	GCAGGCATGATGTAGTCCCA	GCACGGATAGGTACACCACC
NC24	NC-offspring-23	Bomo_Chr14	2192356	C	T	42	26	16	0.38	intron	OUT	F	GGGGAGTCGTTTAGTGGGTG	TCGCATGGATCGTCTGGAAG
NC25	NC-offspring-19	Bomo_Chr17	2432066	G	A	39	24	15	0.38	intergenic	OUT	T	TGGCGGAAGTGGACTTATGG	TTAGTCACCTCGAAGGCGTG
NC26	NC-offspring-14	Bomo_Chr6	16129500	A	T	39	24	15	0.38	intergenic	OUT	T	TTTACGACAACCACGGGGAG	TGACCACAATCCTCGGCTTC
NC27	NC-offspring-27	Bomo_Chr12	16426416	A	G	36	22	14	0.39	intergenic	OUT	T	GAGAGACCGGAAACATGGCA	CGCTAAAACTCGAGAACGGC
NC28	NC-offspring-8	Bomo_Chr13	14215074	C	A	33	20	13	0.39	intergenic	OUT	T	GGAGCGTAGGCTGGAATCTT	GAGAGCCGCACAATACGAGA
NC29	NC-offspring-24	Bomo_Chr10	4254666	C	A	30	18	12	0.40	intron	OUT	T	CGAAAAACAGCATTCGGGCT	TCGTAGGCGTCATAAGGCAC
NC30	NC-offspring-14	Bomo_Chr6	13935671	G	A	45	27	18	0.40	intron	OUT	T	CCACCCTTCAAACCGAAACG	TGACGGAGCTATCCTCGTGT
NC31	NC-offspring-11	Bomo_Chr19	6370594	C	A	32	19	13	0.41	intergenic	OUT	T	TCGTAGCACTTTTGCGGAGT	CGACAAGGGTGCTTGATGGA
NC32	NC-offspring-17	Bomo_Chr7	11757413	C	T	32	19	13	0.41	intergenic	OUT	T	GTCTAAGAAACGCAGCCCAAAC	CAAGCAATGCCTCGACCAAT
NC33	NC-offspring-29	Bomo_Chr4	5281848	C	T	27	16	11	0.41	intergenic	OUT	T	CCGTCAACGAGTTGTCTCCA	CGAGGTCACGAACCACTCAA
NC34	NC-offspring-6	Bomo_Chr11	15169940	A	C	38	22	16	0.42	exon	OUT	T	CTTTGTTCTGGGCGTGTTCG	GGGGACTTATAGCCGGGAGA
NC35	NC-offspring-21	Bomo_Chr16	13183326	C	A	26	15	11	0.42	intron	OUT	T	GCGGTATGATGGCCCTGTAA	AGCTGTTTCCATCTCGTCCG
NC36	NC-offspring-14	Bomo_Chr3	6656473	G	C	33	19	14	0.42	intergenic	IN	T	GACTTGGCTACGCTCCTTCT	AATTCACCCACCTGCCGTAA
NC37	NC-offspring-18	Bomo_Chr1	4351349	A	T	14	8	6	0.43	intron	OUT	F	AAACCGTCGTTAAAGGCCCA	CTGCAAAGAGTTCGACGTGC
NC38	NC-offspring-30	Bomo_Chr13	11037414	C	T	37	21	16	0.43	intergenic	OUT	T	CCGATAAGCCTCCTTCGCAA	CGGCTTTGTCACGCAAACTG
NC39	NC-offspring-12	Bomo_Chr13	5314947	T	A	34	19	15	0.44	intron	OUT	T	TGGACACGGCTAGCTTAACG	CGCACAGACGATAATTGCGG
NC40	NC-offspring-1	Bomo_Chr8	3492258	G	A	36	20	16	0.44	intergenic	OUT	T	GTCCAAAGAAAGGTTGAGGGTA	AGTAATGCTTCGCAGAATCGTA
NC41	NC-offspring-29	Bomo_Chr3	4134264	T	C	42	23	19	0.45	exon	OUT	T	TATTGGGCTTTGCTGAACCT	ATTGAGCGTGGCTTCATTTC
NC42	NC-offspring-28	Bomo_Chr6	4515432	A	T	50	27	23	0.46	intron	OUT	T	GACGTGTTAGGCTGGTCCTC	TCAGGTAATTACGGTGGGCG
NC43	NC-offspring-17	Bomo_Chr24	5135557	A	T	26	14	12	0.46	intergenic	OUT	T	ATTTGGTAGTGCTGTGGCTTAGA	GCTTCATCAGGACAGGTGGG
NC44	NC-offspring-15	Bomo_Chr1	6410731	T	A	30	16	14	0.47	intron	OUT	T	TCGCTAACGAGCACTAAGACC	CAGAGACTGATCTCGCTCGC
NC45	NC-offspring-13	Bomo_Chr3	7194205	C	T	30	16	14	0.47	exon	OUT	T	CGTACACCACGAGCCATAGG	TCAAGACGGGCGTTCAATCA
NC46	NC-offspring-21	Bomo_Chr8	14707532	T	A	47	25	22	0.47	intron	OUT	T	CCTAATATCGTACAAGAGCCTTCATC	ACAGGTGACCGCTTACACGAT
NC47	NC-offspring-9	Bomo_Chr10	10266170	T	A	32	17	15	0.47	intergenic	IN	T	ACATTCGGACCACAAGGAGAC	CGATCGAGTCCGCTTCCTAC
NC48	NC-offspring-12	Bomo_Chr26	2681149	G	A	49	26	23	0.47	intergenic	OUT	T	GCCACGACGACTTACTAACTACCT	GAATGCCGTCAGCCACCTC
NC49	NC-offspring-18	Bomo_Chr21	1687204	C	T	36	19	17	0.47	intron	OUT	T	GGGGCATCTTCCGATACGTT	TTTCCCACTCGCTACCATCG
NC50	NC-offspring-18	Bomo_Chr13	9471056	T	C	40	21	19	0.48	intergenic	OUT	T	AACCTCATTCGTCTGCGACT	TTTAAGGCTCACACCGGACG
NC51	NC-offspring-27	Bomo_Chr16	11790607	A	G	40	21	19	0.48	intergenic	OUT	T	CCATACTGGTAGGTACGCGG	TCTGGACCGACCTCCAAAGA
NC52	NC-offspring-19	Bomo_Chr20	854321	C	T	44	23	21	0.48	intergenic	OUT	T	GACGCCCAAGAATAGGCAGA	CACCGTTAGTGGATCGAGGG
NC53	NC-offspring-18	Bomo_Chr8	15166412	G	A	25	13	12	0.48	intergenic	OUT	T	TTTGGACACTGGAATCGGCA	CAGTGCTCCATGACTTGCCT
NC54	NC-offspring-4	Bomo_Chr11	3984547	T	C	27	14	13	0.48	intergenic	IN	T	ATAGTGTGCTGGTTGCGACA	GCCGGTGCATTGTTATCGAC
NC55	NC-offspring-12	Bomo_Chr1	12012967	T	C	29	15	14	0.48	intergenic	OUT	T	TCGCAATGGAGACCAGAACA	GTGGGTTCAGACAACGAATGC
NC56	NC-offspring-1	Bomo_Scaf001	260233	T	A	31	16	15	0.48	intron	OUT	T	TTCCCGATGGTGATTGGACA	GAGACGGCAACACACGAAAC
NC57	NC-offspring-16	Bomo_Chr10	8935930	A	G	39	20	19	0.49	intron	OUT	T	AAGACTAAGGCAATAGACCCACT	ATGTAGGAACCATAGATCCCAAT
NC58	NC-offspring-4	Bomo_Chr17	2768643	T	C	43	22	21	0.49	intergenic	OUT	T	CAGATCCAGACCATGCACCT	AAGTACGATACGACCCGTGC
NC59	NC-offspring-19	Bomo_Chr2	885244	G	A	34	17	17	0.50	intergenic	OUT	T	ATTATCATAGGCGCGTCCCC	GCCTGTATGACCAGGAAGGG
NC60	NC-offspring-1	Bomo_Chr7	12194406	T	C	30	15	15	0.50	intergenic	OUT	T	TCTGCGCGTCTACGATTCAG	CGAGTCTTCGCCGACCATAA
NC61	NC-offspring-4	Bomo_Chr25	12651239	G	A	38	19	19	0.50	intergenic	IN	T	GCGCTTCATGCAAGAGATGG	CGAGGCAGTGGAAGGAAAGT
NC62	NC-offspring-4	Bomo_Chr21	6637923	C	A	36	18	18	0.50	intergenic	IN	T	AAACTCGGTACGAAACGGGC	GATTCGCCGGCCTGACTATAA

NC63	NC-offspring-10	Bomo_Chr20	2502150	T	C	26	13	13	0.50	intron	OUT	T	GTTCCTTCTCGTTGAGCCAGTT	GTACTAAGCGGTGCGTATCCC
NC64	NC-offspring-30	Bomo_Chr14	2282655	G	C	35	17	18	0.51	intron	OUT	T	TGCCTCTTGTGTCGGAATGT	TTTGCGGAGACTCACGGTTA
NC65	NC-offspring-23	Bomo_Chr8	15724617	T	C	31	15	16	0.52	intron	OUT	T	CTTTTCGTCGCCCGATCAAC	ATAGACACACACAGCGAGCC
NC66	NC-offspring-13	Bomo_Chr28	3227528	T	C	25	12	13	0.52	intron	OUT	T	GAACACGTTCCGTCCAGTGT	ATCAAAGCCGGCAATGTGAC
NC67	NC-offspring-28	Bomo_Chr2	791699	A	G	38	18	20	0.53	intergenic	IN	T	CGTTCGCTTTTCGAGTCAGC	TTGGCGGTTTTGGAATGAGC
NC68	NC-offspring-2	Bomo_Chr12	8870076	C	T	38	18	20	0.53	intergenic	IN	T	GTACTCAGTGCCAGCGACTT	ACATCAGCCCGGACGAATAC
NC69	NC-offspring-11	Bomo_Chr12	4561504	G	A	26	12	14	0.54	intergenic	OUT	T	TATGCGTGCATGCGACATTG	AGCTTCGTCCATCTAAGCCG
NC70	NC-offspring-2	Bomo_Chr21	3259305	C	T	73	33	40	0.55	intergenic	OUT	T	AGTGAGGTATCGGCTATTATCGTA	TCTACTGGTGATAGGACCTGTTGT
NC71	NC-offspring-8	Bomo_Chr18	7981971	G	A	18	8	10	0.56	intergenic	IN	T	CGCCTCGTTGATAGTGGTTTG	AACTCGTATGACTGTTTCGCCTC
NC72	NC-offspring-4	Bomo_Chr25	1203868	G	A	39	17	22	0.56	intergenic	OUT	T	GCTATGCCAGGTTCACCAAGT	AGCTTCCCACTTCGCATTCT
NC73	NC-offspring-22	Bomo_Chr17	7615541	A	T	30	13	17	0.57	intron	OUT	T	AGTGTGACTGAAATGCCTACCG	GGTGTCAGGAAGGGAAGGAGA
NC74	NC-offspring-1	Bomo_Chr23	3610379	C	A	27	11	16	0.59	intergenic	OUT	T	CTATTCTCGGCTGCGTGGAT	CCGGAACATCAAGAGCACCT
NC75	NC-offspring-16	Bomo_Chr24	6121645	A	T	30	12	18	0.60	intergenic	OUT	T	AGTACCCGGATTTCGAAGCG	CCGTGTGTGCTCACCAAATG
NC76	NC-offspring-14	Bomo_Chr9	14438522	T	A	48	19	29	0.60	intron	IN	T	CGTAGATGATACACGCAAGGC	TCTAGCCCGAATTAGTCTAAAGGA
NC77	NC-offspring-3	Bomo_Chr7	12422408	T	A	32	12	20	0.63	intron	OUT	T	ATTGGGACCACAAATAGAGCG	CGAATACGAATCGGTAATAAGACA
NC78	NC-offspring-15	Bomo_Chr18	13580932	G	A	34	12	22	0.65	intergenic	OUT	T	CGGCTGTCACGATACAAGTCC	TGGCCGAGCAGGCAGAC
NC79	NC-offspring-27	Bomo_Chr4	7639783	C	T	40	14	26	0.65	intron	OUT	T	TCGTTAGTGCGCTACTGCAT	AAAGTAGCCCTGACTGCACC
NC80	NC-offspring-10	Bomo_Chr3	4906455	T	A	26	9	17	0.65	intergenic	OUT	T	TGAATCACGCGGACCGAAA	GGAATTGAGCACCACAACGG
NC81	NC-offspring-2	Bomo_Chr27	7801770	A	C	38	13	25	0.66	intergenic	OUT	T	GGGCTGTGTATGTGGGCTAA	TACCCTCTCGGGTTGAGTCC
NC82	NC-offspring-19	Bomo_Chr1	16978349	T	A	21	7	14	0.67	intergenic	OUT	T	TGCGTGCGTAGATACCTGATTC	ACTATACTGCCAATCGTGAACTTGT
NC83	NC-offspring-3	Bomo_Chr4	10820685	C	T	38	11	27	0.71	intron	OUT	T	TTAAGCTGATTGGCCGCAGA	GATCGTTCTCAATGCGTGCC
NC84	NC-offspring-13	Bomo_Chr3	9909934	A	T	22	6	16	0.73	intron	OUT	T	ATCACTTTGAGAAGGCGGCA	ACGAGGCCGGTCCTAGTAAT
NC85	NC-offspring-11	Bomo_Chr12	16123796	G	A	45	27	18	0.40	intergenic	IN	no band	GTACCACCGCCTTGCCTATT	GCGTCGTATTCCTCAGTGCT
NC86	NC-offspring-18	Bomo_Chr23	1606236	A	G	42	24	18	0.43	intergenic	OUT	no band	CTGTCGTGTTGTGTTTCGGG	CCTTCTCAGTGAGTCGCGTT
NC87	NC-offspring-10	Bomo_Chr9	15515750	C	A	22	12	10	0.45	intron	OUT	no band	AGCTCACTAGCACCGACAAG	AGCTCACTAGCACCGACAAG
NC88	NC-offspring-26	Bomo_Chr5	16064276	T	A	51	26	25	0.49	intergenic	OUT	no band	CTTTCCACGCATGTGGGTTT	GCCTCCGAAGGAGACCTAAC
NC89	NC-offspring-26	Bomo_Chr3	7669184	G	A	33	16	17	0.52	intron	OUT	no band	TTCTTGTGAGTCTGCACGGG	TTTCATACTCCTCCGTCCGC
NC90	NC-offspring-1	Bomo_Chr15	14236033	T	C	19	9	10	0.53	intron	IN	no band	TGCTTCGGACGTCTTATCCG	AAGATTTGAACCGTCGGCCT
NC91	NC-offspring-8	Bomo_Chr17	2179918	A	G	34	16	18	0.53	intron	OUT	no band	TGACTATTGCGCTGAGGGTC	ACGGCCCACCTGATGTTAAG
NC92	NC-offspring-2	Bomo_Chr2	5285664	A	T	32	15	17	0.53	intergenic	IN	no band	TTGGGTGGGTGAGAAAGACG	AAGCACGCAGCGAAGAAAAC
NC93	NC-offspring-15	Bomo_Chr17	287866	T	G	15	7	8	0.53	exon	IN	no band	GTACCTCGGCCTCATTCTGG	GGGGGACCCGAAACACTTAG
NC94	NC-offspring-25	Bomo_Chr21	14217853	T	C	30	14	16	0.53	intergenic	OUT	no band	CCTCTGGTAGCCAATTAACACC	ACCGTGCCTCCTTCATTCTA
NC95	NC-offspring-17	Bomo_Chr8	14489965	A	G	27	12	15	0.56	intron	OUT	no band	TGACCTCTTTGCCCAGCAAT	CTACCATCAGGGCGTAGCTG
NC96	NC-offspring-11	Bomo_Chr8	10770419	G	A	39	17	22	0.56	exon	OUT	no band	GACCCCCTTGTGTAGTGACC	TGATGGGTGCGACACTCCTA
NC97	NC-offspring-11	Bomo_Chr1	229385	A	C	44	19	25	0.57	intron	OUT	no band	GCAAGCGTACTTTTGGGAGC	GATGGGTGCGACACTCCTAC
NC98	NC-offspring-10	Bomo_Chr20	10039975	T	A	21	9	12	0.57	intergenic	OUT	no band	AGGGCTCTCGAAATGTGGTG	CCGCGTGAAGCTGAAATAGC
NC99	NC-offspring-21	Bomo_Chr21	4597013	G	T	33	14	19	0.58	intergenic	OUT	no band	CCACAAAGAGGCAACGCAAA	GCAACAGCCTAGAGCCAAGA
NC100	NC-offspring-23	Bomo_Chr1	5641333	G	A	17	7	10	0.59	intron	OUT	no band	GTGTGCGTCAAATGCATGGT	GATGACGACGGGTTCGCTAT
NC101	NC-offspring-2	Bomo_Chr4	16350687	C	T	34	14	20	0.59	intergenic	IN	no band	CAATCTTGCCTGTTTCCGCC	GACCTTACCTCGCTGGTGAC
NC102	NC-offspring-18	Bomo_Chr16	144208	T	C	37	15	22	0.59	intergenic	OUT	no band	CGACAAAGTCTGGCACAACG	GCGGCAACAATAAGGCGATT
NC103	NC-offspring-4	Bomo_Chr5	16878039	G	A	38	14	24	0.63	intergenic	IN	no band	GCAATCACAGCGATTCGGTT	TAGGTGGTATACGCGTTGCG
NC104	NC-offspring-23	Bomo_Chr24	10406786	T	C	42	15	27	0.64	intergenic	IN	no band	GAATCGAACCCACGACCCTC	GCGAGCTCGTTCAACCATTT
NC105	NC-offspring-21	Bomo_Chr9	4004163	T	C	27	7	20	0.74	intergenic	OUT	no band	TGGGTGGTCGAGCATTTGTT	GTGCCCTTAGAGTTTCGGCT
NC106	NC-offspring-23	Bomo_Chr2	7047647	C	T	30	14	16	0.53	intron	OUT	\	\	\

High temperature

Variant Numbe	Sample	Chromosome	Position	Ref	Alt	Read depth	Ref reads	Alt reads	Proportion of Alt read	Region		Validation	Forward primer	Reverse primer
HT1	HT-offspring-6	Bomo_Chr22	12045005	C	G	23	12	11	0.48	intergenic	OUT	T	GGTGGGATTCCAGTGTTGGT	TATTCGTAGCGACACCTGCC
HT2	HT-offspring-8	Bomo_Chr23	3704124	G	A	33	15	18	0.55	intergenic	OUT	T	AGAGCAGGAGCTAGGTGTCT	AACGGCTGATGGGGCTAAAA
HT3	HT-offspring-10	Bomo_Chr23	19858453	G	T	29	18	11	0.38	intergenic	OUT	T	TGAGGATACGGCCCACCTAA	TTCGAACCCCATCACAGTCG
HT4	HT-offspring-10	Bomo_Chr24	1666203	T	C	18	11	7	0.39	intergenic	OUT	T	TCCTTCGTTTTCACTAGGCCC	ATGCGCTACACGGATCAAGT
HT5	HT-offspring-10	Bomo_Chr26	6715071	G	C	29	11	18	0.62	intergenic	OUT	T	AAAACCCCGAGATGGGTGAC	GGCGCCAATGTGGTTTCTTT
HT6	HT-offspring-6	Bomo_Chr27	10613054	T	G	17	5	12	0.71	intergenic	OUT	T	GAATGTCTTTTGGTCGGCGG	TTGTTTGGTCCTCCGTGGC
HT7	HT-offspring-3	Bomo_Chr28	3126377	A	G	30	17	13	0.43	intergenic	IN	T	TAGACAGAGCGGATCACACG	CGGTCAATGTCTCTGTCGCT
HT8	HT-offspring-1	Bomo_Chr4	7150454	C	G	11	6	5	0.45	intron	IN	T	ACACCCTTTTCACCGTGGTT	GTTACAGCGGATCAGTGGCT
HT9	HT-offspring-8	Bomo_Chr5	3600521	C	A	23	10	13	0.57	intergenic	OUT	T	GTGGCGTTGAAGAGTACCGA	TGGGTGAGGTGTTTATGGGC
HT10	HT-offspring-10	Bomo_Chr7	8771502	C	G	27	17	10	0.37	intergenic	OUT	T	CCCGCTCGCCAATTCACTAA	CCCTTTGTTTAAGCCGTCGC
HT11	HT-offspring-2	Bomo_Chr7	11587262	C	T	24	13	11	0.46	intergenic	IN	T	TAAGCATCGAAGACCGACCG	TGCCACCTCGTATTGCTTGT
HT12	HT-offspring-2	Bomo_Chr9	16700363	G	A	22	14	8	0.36	intergenic	OUT	T	TGGAGGCACATCTAATGCGT	TAATTGCAGCGTCAAGTGCG
HT13	HT-offspring-8	Bomo_Chr11	20196424	C	T	22	12	10	0.45	intergenic	OUT	T	GTAACACCGCACGTTCACAG	GAGCCCGAGAAGCTAAGTCC
HT14	HT-offspring-4	Bomo_Chr12	6195345	C	T	31	12	19	0.61	intergenic	OUT	T	TGCCAATTCCGTGGAAGTCA	GAAAGTTCCCCTGCGGCTAT
HT15	HT-offspring-3	Bomo_Chr16	13563717	A	T	23	7	16	0.70	intergenic	OUT	T	AAGCTGGTCCGACTTCTGTG	ACTTCTTTGTAGAAGCGGCG
HT16	HT-offspring-3	Bomo_Chr17	11294215	G	T	18	6	12	0.67	intergenic	OUT	T	ATGACTGTGGTCCCCGAAAC	CTTTGTGTGGCCATTTGCGA
HT17	HT-offspring-2	Bomo_Chr18	11501635	C	T	25	14	11	0.44	intergenic	OUT	T	CGACCCGCATTTACTGACGA	ATTACACTTGTTGGGGCTGGA
HT18	HT-offspring-1	Bomo_Chr20	11157672	G	A	21	8	13	0.62	intron	OUT	T	GCTCGTCTGCCTACAAGGTT	ATGCGAAGTGATGGAGAGCA
HT19	HT-offspring-3	Bomo_Chr22	9566002	T	C	24	12	12	0.50	exon	OUT	T	AGTCTTGTTCCGCCCGTTT	TTGGCAAGTACTCGTGTTTTTCT

HT20	HT-offspring-7	Bomo_Chr22	12838456	G	A	18	11	7	0.39	intergenic	OUT	T	TGGGAAAGTGTACATCGGGC	GTGTTTTGAACAGAGCGCGT
HT21	HT-offspring-4	Bomo_Chr22	4397406	C	G	13	7	6	0.46	intron	OUT	\	\	\
HT22	HT-offspring-4	Bomo_Chr15	15967075	T	C	26	14	12	0.46	intergenic	OUT	\	\	\
HT23	HT-offspring-7	Bomo_Chr7	8002153	T	C	29	20	9	0.31	intergenic	OUT	\	\	\
HT24	HT-offspring-3	Bomo_Chr8	15308025	G	A	16	11	5	0.31	intergenic	OUT	\	\	\
HT25	HT-offspring-3	Bomo_Chr8	13704263	G	A	19	13	6	0.32	intergenic	IN	\	\	\
HT26	HT-offspring-1	Bomo_Chr27	9944202	A	G	22	15	7	0.32	intergenic	OUT	\	\	\
HT27	HT-offspring-5	Bomo_Chr11	18663809	C	T	18	12	6	0.33	intergenic	OUT	\	\	\
HT28	HT-offspring-4	Bomo_Chr17	13226651	C	A	26	17	9	0.35	exon	OUT	\	\	\
HT29	HT-offspring-3	Bomo_Chr3	2850560	G	T	23	15	8	0.35	intergenic	OUT	\	\	\
HT30	HT-offspring-8	Bomo_Chr6	7705702	G	A	14	9	5	0.36	intron	OUT	\	\	\
HT31	HT-offspring-3	Bomo_Chr9	11669641	C	T	21	13	8	0.38	intron	OUT	\	\	\
HT32	HT-offspring-5	Bomo_Chr15	11572664	A	C	24	14	10	0.42	intron	OUT	\	\	\
HT33	HT-offspring-10	Bomo_Chr4	11652949	A	T	24	14	10	0.42	intergenic	IN	\	\	\
HT34	HT-offspring-6	Bomo_Chr15	1468256	A	C	21	12	9	0.43	intron	OUT	\	\	\
HT35	HT-offspring-8	Bomo_Chr18	10055768	T	C	15	8	7	0.47	intron	OUT	\	\	\
HT36	HT-offspring-4	Bomo_Chr11	13617206	T	G	15	8	7	0.47	intergenic	OUT	\	\	\
HT37	HT-offspring-9	Bomo_Chr21	12202293	A	G	27	14	13	0.48	intergenic	OUT	\	\	\
HT38	HT-offspring-6	Bomo_Chr13	1861996	T	C	29	15	14	0.48	intron	OUT	\	\	\
HT39	HT-offspring-2	Bomo_Chr24	435531	A	T	6	3	3	0.50	intergenic	OUT	\	\	\
HT40	HT-offspring-7	Bomo_Chr10	16014341	A	T	20	10	10	0.50	intron	OUT	\	\	\
HT41	HT-offspring-9	Bomo_Chr10	11989876	G	A	21	10	11	0.52	intergenic	IN	\	\	\
HT42	HT-offspring-8	Bomo_Chr8	14876585	G	A	28	13	15	0.54	intron	IN	\	\	\
HT43	HT-offspring-3	Bomo_Chr23	10585292	A	G	18	8	10	0.56	intron	OUT	\	\	\
HT44	HT-offspring-7	Bomo_Chr24	4246628	C	T	19	8	11	0.58	intergenic	OUT	\	\	\
HT45	HT-offspring-10	Bomo_Chr13	3434412	G	T	17	7	10	0.59	intergenic	OUT	\	\	\
HT46	HT-offspring-8	Bomo_Chr1	10411614	G	A	22	8	14	0.64	intergenic	IN	\	\	\
HT47	HT-offspring-8	Bomo_Chr20	5765713	A	G	29	10	19	0.66	intergenic	OUT	\	\	\
HT48	HT-offspring-5	Bomo_Chr10	7468077	A	G	20	3	17	0.85	exon	OUT	\	\	\

Low temperature

Variant Number	Sample	Chromesome	Position	Ref	Alt	Read depth	Ref reads	Alt reads	Proportion of Alt reads	Region		Validation	Forward primer	Reverse primer
LT1	LT-offspring-5	Bomo_Chr25	2452472	A	C	20	11	9	0.45	intergenic	OUT	T	GACGGGCGATTGTTTAGTGC	GTAAGAGTGCAGGGGAGTCG
LT2	LT-offspring-8	Bomo_Chr5	17207321	C	A	23	12	11	0.48	intergenic	OUT	T	ACATTACCGCCATTAGGGCA	TGGAAAGCTGCCGTCGTTAT
LT3	LT-offspring-7	Bomo_Chr28	8706402	G	A	25	13	12	0.48	intergenic	IN	T	CCTCCTGAAGCCAGATGGTG	CATCACCCTATCCTCCCTCG
LT4	LT-offspring-6	Bomo_Chr26	11333293	G	T	20	10	10	0.50	intergenic	OUT	T	GCGACAAGGTTCAGCAACTC	GGTTTCGTTCCCAACCCTCT
LT5	LT-offspring-8	Bomo_Chr14	3421776	C	T	25	12	13	0.52	intergenic	OUT	T	GGGGAAACCCCTCTGCCT	TCCCGCCAAAAAGTCCCTG
LT6	LT-offspring-6	Bomo_Chr4	7550538	T	A	23	11	12	0.52	intergenic	OUT	T	CCGTATGCTGGTTCGTCTGT	TGCTGGTTATTCGTGACCGA
LT7	LT-offspring-2	Bomo_Chr21	10057248	G	T	27	12	15	0.56	intergenic	OUT	T	GCTTGCCGGTCATTTTACCC	GCCCTGTCGTGAATACTTCG
LT8	LT-offspring-5	Bomo_Chr17	10028944	G	A	40	17	23	0.58	intron	IN	T	CGGGGTGATCACACATTCCA	TGTGGGCGGTATGTAACTGC
LT9	LT-offspring-9	Bomo_Chr15	4949810	A	G	27	11	16	0.59	intergenic	OUT	T	GGTCCGTTGGCAGATACCTC	GTGCTGAAAGCATAAGCGGC
LT10	LT-offspring-6	Bomo_Chr11	11830078	G	A	20	8	12	0.60	exon	OUT	T	CGGAGTACAGTTAGCGCAGG	GAACTGTTTGGCACTGCTATTC
LT11	LT-offspring-7	Bomo_Chr16	5281095	C	T	22	5	17	0.77	intergenic	OUT	T	CGTTTCAGAAGCTCCAGTGC	CGGCTTATCTGATGTTCGGT
LT12	LT-offspring-3	Bomo_Chr17	14176597	G	T	17	11	6	0.35	intron	OUT	T	GTGTGGTGACATCGAGACGA	GCCATCTTAGCATCGACGTG
LT13	LT-offspring-8	Bomo_Chr20	6457627	G	T	25	16	9	0.36	intron	OUT	T	ACTGGCGGGGTGTTCTTATG	GCTTTCCAGAGCCCAATCCT
LT14	LT-offspring-8	Bomo_Chr11	17195085	C	G	22	14	8	0.36	intron	OUT	T	TACCACAAGCATCGTCCTCG	CGTCATTAAGTCCGCACACG
LT15	LT-offspring-1	Bomo_Chr25	9833409	G	A	13	8	5	0.38	intron	OUT	T	ATCAGCGAGCCTTGAGTACG	GTTGCCGAAGATCATGTCGC
LT16	LT-offspring-1	Bomo_Chr23	13500367	A	T	13	8	5	0.38	intergenic	IN	T	TCTGTGGGCCGCATTCTAAG	CGCTTGGGTAAGCATCAACG
LT17	LT-offspring-10	Bomo_Chr10	11837225	G	A	18	11	7	0.39	intergenic	OUT	T	CTAGTCAAAACCGGCCCACT	TTGGTTCCAGGAGTCGCATC
LT18	LT-offspring-8	Bomo_Chr25	2872459	C	A	23	14	9	0.39	intergenic	OUT	T	GAGGATCGTTCATTGGCGGA	CGGCTTAATTGTCACGGCAC
LT19	LT-offspring-1	Bomo_Chr15	16941965	T	G	22	13	9	0.41	intron	OUT	T	AAAGGCGGACTGCCATTACT	TTCCACGCGCTTAATAGCCA
LT20	LT-offspring-4	Bomo_Chr10	15881638	T	C	29	17	12	0.41	exon	OUT	T	ACTAACCCTGAAGCCGAAGC	CTCGGACACCTCGTCGTATG
LT21	LT-offspring-6	Bomo_Chr13	1457891	C	T	12	7	5	0.42	intergenic	IN	\	\	\
LT22	LT-offspring-1	Bomo_Chr12	7528199	G	A	30	17	13	0.43	intergenic	OUT	\	\	\
LT23	LT-offspring-5	Bomo_Chr3	212920	A	T	20	14	6	0.30	intergenic	OUT	\	\	\
LT24	LT-offspring-6	Bomo_Chr4	17564731	C	T	20	14	6	0.30	intergenic	OUT	\	\	\
LT25	LT-offspring-2	Bomo_Chr9	16563226	C	A	19	13	6	0.32	intron	OUT	\	\	\
LT26	LT-offspring-7	Bomo_Chr7	12061125	A	G	22	15	7	0.32	intron	OUT	\	\	\
LT27	LT-offspring-8	Bomo_Chr9	12498027	C	T	22	15	7	0.32	intergenic	OUT	\	\	\
LT28	LT-offspring-7	Bomo_Chr2	6725532	A	T	25	17	8	0.32	intergenic	OUT	\	\	\
LT29	LT-offspring-4	Bomo_Chr6	4398465	G	T	23	15	8	0.35	intergenic	OUT	\	\	\
LT30	LT-offspring-8	Bomo_Chr4	7688387	C	T	11	7	4	0.36	intergenic	OUT	\	\	\
LT31	LT-offspring-2	Bomo_Chr4	3879418	T	C	15	9	6	0.40	intergenic	OUT	\	\	\
LT32	LT-offspring-9	Bomo_Chr9	9965125	A	G	22	13	9	0.41	intergenic	OUT	\	\	\
LT33	LT-offspring-10	Bomo_Chr25	4214457	A	C	12	7	5	0.42	intergenic	OUT	\	\	\
LT34	LT-offspring-4	Bomo_Chr12	12910891	C	A	24	14	10	0.42	intergenic	OUT	\	\	\

Supplementary table S2 continue

LT35	LT-offspring-3	Bomo_Chr12	10113395	C	A	28	16	12	0.43	intergenic	OUT	\	\	\
LT36	LT-offspring-2	Bomo_Chr28	8398364	G	A	25	14	11	0.44	intron	OUT	\	\	\
LT37	LT-offspring-6	Bomo_Chr5	16870856	G	A	18	10	8	0.44	intergenic	IN	\	\	\
LT38	LT-offspring-1	Bomo_Chr7	2632017	A	T	29	16	13	0.45	intergenic	IN	\	\	\
LT39	LT-offspring-9	Bomo_Chr4	8346938	T	C	11	6	5	0.45	intron	OUT	\	\	\
LT40	LT-offspring-10	Bomo_Chr16	9404338	T	C	22	11	11	0.50	intron	OUT	\	\	\
LT41	LT-offspring-6	Bomo_Chr25	11619771	T	C	14	7	7	0.50	exon	OUT	\	\	\
LT42	LT-offspring-5	Bomo_Chr10	12061810	A	G	18	9	9	0.50	intergenic	OUT	\	\	\
LT43	LT-offspring-6	Bomo_Chr25	13991804	T	A	18	9	9	0.50	intergenic	OUT	\	\	\
LT44	LT-offspring-8	Bomo_Chr22	3454265	C	T	26	12	14	0.54	intergenic	OUT	\	\	\
LT45	LT-offspring-7	Bomo_Chr16	2634170	C	T	23	10	13	0.57	intergenic	OUT	\	\	\
LT46	LT-offspring-6	Bomo_Chr27	9736194	T	C	19	8	11	0.58	intergenic	OUT	\	\	\
LT47	LT-offspring-7	Bomo_Chr4	2457498	T	C	11	4	7	0.64	intron	OUT	\	\	\

Supplementary table S3. The callable sites, *de novo* mutations and mutation rate of A/T bases and C/G bases of each genome.

Normal condition				
**Samples**	**Type**	**Callable sites**	**DNMs**	**Mutation rate**
NC-offspring-1	A/T	268958939	3	5.58E-09
	C/G	174789627	2	5.72E-09
NC-offspring-2	A/T	269200386	2	3.71E-09
	C/G	174963584	3	8.57E-09
NC-offspring-3	A/T	268978318	1	1.86E-09
	C/G	174805058	1	2.86E-09
NC-offspring-4	A/T	268993707	2	3.72E-09
	C/G	174814567	4	1.14E-08
NC-offspring-5	A/T	269084915	0	0.00E+00
	C/G	174878885	0	0.00E+00
NC-offspring-6	A/T	269114062	2	3.72E-09
	C/G	174898390	0	0.00E+00
NC-offspring-7	A/T	269030733	0	0.00E+00
	C/G	174844270	1	2.86E-09
NC-offspring-8	A/T	268625491	1	1.86E-09
	C/G	174556031	2	5.73E-09
NC-offspring-9	A/T	269124370	3	5.57E-09
	C/G	174907598	0	0.00E+00
NC-offspring-10	A/T	268780770	3	5.58E-09
	C/G	174662354	1	2.86E-09
NC-offspring-11	A/T	269141698	1	1.86E-09
	C/G	174920238	4	1.14E-08
NC-offspring-12	A/T	269005495	3	5.58E-09
	C/G	174823757	1	2.86E-09
NC-offspring-13	A/T	268407932	4	7.45E-09
	C/G	174404284	4	1.15E-08
NC-offspring-14	A/T	269145423	2	3.72E-09
	C/G	174921096	2	5.72E-09
NC-offspring-15	A/T	268979652	3	5.58E-09
	C/G	174805945	1	2.86E-09
NC-offspring-16	A/T	268752876	3	5.58E-09
	C/G	174681921	0	0.00E+00
NC-offspring-17	A/T	268345513	2	3.73E-09
	C/G	174427833	1	2.87E-09
NC-offspring-18	A/T	268921117	4	7.44E-09
	C/G	174788996	3	8.58E-09
NC-offspring-19	A/T	268929646	1	1.86E-09
	C/G	174794804	3	8.58E-09
NC-offspring-20	A/T	268687451	1	1.86E-09
	C/G	174641563	0	0.00E+00
NC-offspring-21	A/T	269022366	3	5.58E-09
	C/G	174854186	2	5.72E-09
NC-offspring-22	A/T	268650981	2	3.72E-09
	C/G	174620880	0	0.00E+00
NC-offspring-23	A/T	269108936	3	5.57E-09
	C/G	174909819	3	8.58E-09
NC-offspring-24	A/T	268556123	1	1.86E-09
	C/G	174557512	2	5.73E-09
NC-offspring-25	A/T	268054890	1	1.87E-09
	C/G	174247081	0	0.00E+00
NC-offspring-26	A/T	268933941	1	1.86E-09
	C/G	174798600	1	2.86E-09

Supplementary table S3 continue

NC-offspring-27	A/T	268994300	3	5.58E-09
	C/G	174835925	1	2.86E-09
NC-offspring-28	A/T	269050711	2	3.72E-09
	C/G	174868715	1	2.86E-09
NC-offspring-29	A/T	268513143	1	1.86E-09
	C/G	174528449	2	5.73E-09
NC-offspring-30	A/T	269015363	0	0.00E+00
	C/G	174848604	3	8.58E-09
**High temperature**				
**Samples**	**Type**	**Callable sites**	**DNMs**	**Mutation rate**
HT-offspring-1	A/T	265048089	1	1.89E-09
	C/G	172554609	2	5.80E-09
HT-offspring-2	A/T	261746005	1	1.91E-09
	C/G	170466498	3	8.80E-09
HT-offspring-3	A/T	261772711	4	7.64E-09
	C/G	170486422	5	1.47E-08
HT-offspring-4	A/T	263335186	2	3.80E-09
	C/G	171469721	3	8.75E-09
HT-offspring-5	A/T	252798370	2	3.96E-09
	C/G	164779421	1	3.03E-09
HT-offspring-6	A/T	267105816	3	5.62E-09
	C/G	173746991	1	2.88E-09
HT-offspring-7	A/T	265101785	2	3.77E-09
	C/G	172443584	2	5.80E-09
HT-offspring-8	A/T	267483994	2	3.74E-09
	C/G	173971981	6	1.72E-08
HT-offspring-9	A/T	267169550	1	1.87E-09
	C/G	173811460	1	2.88E-09
HT-offspring-10	A/T	265606109	2	3.76E-09
	C/G	172811499	4	1.16E-08
**Low temperature**				
**Samples**	**Type**	**Callable sites**	**DNMs**	**Mutation rate**
LT-offspring-1	A/T	265699268	3	5.65E-09
	C/G	172958036	2	5.78E-09
LT-offspring-2	A/T	262779672	1	1.90E-09
	C/G	171127905	3	8.77E-09
LT-offspring-3	A/T	257273764	0	0.00E+00
	C/G	167625386	2	5.97E-09
LT-offspring-4	A/T	263784094	1	1.90E-09
	C/G	171776500	2	5.82E-09
LT-offspring-5	A/T	267046686	3	5.62E-09
	C/G	173815896	1	2.88E-09
LT-offspring-6	A/T	265184276	4	7.54E-09
	C/G	172440811	5	1.45E-08
LT-offspring-7	A/T	266902471	3	5.62E-09
	C/G	173667506	3	8.64E-09
LT-offspring-8	A/T	264431329	0	0.00E+00
	C/G	172033565	8	2.33E-08
LT-offspring-9	A/T	267312758	3	5.61E-09
	C/G	173883212	0	0.00E+00
LT-offspring-10	A/T	265638240	2	3.76E-09
	C/G	172788578	1	2.89E-09

Supplementary table S4. The callable sites, *de novo* mutations and mutation rate of each mutation type of each genome.

Normal condition				
**Sample**	**Mutation type**	**Callable sites**	**DNMs**	**Mutation rate**
NC-offspring-1	A:T→C:G	268958939	0	0.00E+00
	A:T→G:C	268958939	2	3.72E-09
	A:T→T:A	268958939	1	1.86E-09
	G:C→A:T	174789627	1	2.86E-09
	G:C→C:G	174789627	0	0.00E+00
	G:C→T:A	174789627	1	2.86E-09
NC-offspring-2	A:T→C:G	269200386	1	1.86E-09
	A:T→G:C	269200386	0	0.00E+00
	A:T→T:A	269200386	1	1.86E-09
	G:C→A:T	174963584	3	8.57E-09
	G:C→C:G	174963584	0	0.00E+00
	G:C→T:A	174963584	0	0.00E+00
NC-offspring-3	A:T→C:G	268978318	0	0.00E+00
	A:T→G:C	268978318	0	0.00E+00
	A:T→T:A	268978318	1	1.86E-09
	G:C→A:T	174805058	1	2.86E-09
	G:C→C:G	174805058	0	0.00E+00
	G:C→T:A	174805058	0	0.00E+00
NC-offspring-4	A:T→C:G	268993707	0	0.00E+00
	A:T→G:C	268993707	2	3.72E-09
	A:T→T:A	268993707	0	0.00E+00
	G:C→A:T	174814567	3	8.58E-09
	G:C→C:G	174814567	0	0.00E+00
	G:C→T:A	174814567	1	2.86E-09
NC-offspring-5	A:T→C:G	269084915	0	0.00E+00
	A:T→G:C	269084915	0	0.00E+00
	A:T→T:A	269084915	0	0.00E+00
	G:C→A:T	174878885	0	0.00E+00
	G:C→C:G	174878885	0	0.00E+00
	G:C→T:A	174878885	0	0.00E+00
NC-offspring-6	A:T→C:G	269114062	1	1.86E-09
	A:T→G:C	269114062	1	1.86E-09
	A:T→T:A	269114062	0	0.00E+00
	G:C→A:T	174898390	0	0.00E+00
	G:C→C:G	174898390	0	0.00E+00
	G:C→T:A	174898390	0	0.00E+00
NC-offspring-7	A:T→C:G	269030733	0	0.00E+00
	A:T→G:C	269030733	0	0.00E+00
	A:T→T:A	269030733	0	0.00E+00
	G:C→A:T	174844270	1	2.86E-09
	G:C→C:G	174844270	0	0.00E+00
	G:C→T:A	174844270	0	0.00E+00
NC-offspring-8	A:T→C:G	268625491	0	0.00E+00
	A:T→G:C	268625491	1	1.86E-09
	A:T→T:A	268625491	0	0.00E+00
	G:C→A:T	174556031	1	2.86E-09
	G:C→C:G	174556031	0	0.00E+00
	G:C→T:A	174556031	1	2.86E-09
NC-offspring-9	A:T→C:G	269124370	1	1.86E-09
	A:T→G:C	269124370	0	0.00E+00
	A:T→T:A	269124370	2	3.72E-09
	G:C→A:T	174907598	0	0.00E+00

Supplementary table S4 continue

	G:C→C:G	174907598	0	0.00E+00
	G:C→T:A	174907598	0	0.00E+00
NC-offspring-10	A:T→C:G	268780770	0	0.00E+00
	A:T→G:C	268780770	1	1.86E-09
	A:T→T:A	268780770	2	3.72E-09
	G:C→A:T	174662354	0	0.00E+00
	G:C→C:G	174662354	0	0.00E+00
	G:C→T:A	174662354	1	2.86E-09
NC-offspring-11	A:T→C:G	269141698	1	1.86E-09
	A:T→G:C	269141698	0	0.00E+00
	A:T→T:A	269141698	0	0.00E+00
	G:C→A:T	174920238	3	8.58E-09
	G:C→C:G	174920238	0	0.00E+00
	G:C→T:A	174920238	1	2.86E-09
NC-offspring-12	A:T→C:G	269005495	0	0.00E+00
	A:T→G:C	269005495	1	1.86E-09
	A:T→T:A	269005495	2	3.72E-09
	G:C→A:T	174823757	1	2.86E-09
	G:C→C:G	174823757	0	0.00E+00
	G:C→T:A	174823757	0	0.00E+00
NC-offspring-13	A:T→C:G	268407932	0	0.00E+00
	A:T→G:C	268407932	3	5.59E-09
	A:T→T:A	268407932	1	1.86E-09
	G:C→A:T	174404284	4	1.15E-08
	G:C→C:G	174404284	0	0.00E+00
	G:C→T:A	174404284	0	0.00E+00
NC-offspring-14	A:T→C:G	269145423	0	0.00E+00
	A:T→G:C	269145423	0	0.00E+00
	A:T→T:A	269145423	2	3.72E-09
	G:C→A:T	174921096	1	2.86E-09
	G:C→C:G	174921096	1	2.86E-09
	G:C→T:A	174921096	0	0.00E+00
NC-offspring-15	A:T→C:G	268979652	2	3.72E-09
	A:T→G:C	268979652	0	0.00E+00
	A:T→T:A	268979652	1	1.86E-09
	G:C→A:T	174805945	1	2.86E-09
	G:C→C:G	174805945	0	0.00E+00
	G:C→T:A	174805945	0	0.00E+00
NC-offspring-16	A:T→C:G	268752876	0	0.00E+00
	A:T→G:C	268752876	2	3.72E-09
	A:T→T:A	268752876	1	1.86E-09
	G:C→A:T	174681921	0	0.00E+00
	G:C→C:G	174681921	0	0.00E+00
	G:C→T:A	174681921	0	0.00E+00
NC-offspring-17	A:T→C:G	268345513	0	0.00E+00
	A:T→G:C	268345513	1	1.86E-09
	A:T→T:A	268345513	1	1.86E-09
	G:C→A:T	174427833	1	2.87E-09
	G:C→C:G	174427833	0	0.00E+00
	G:C→T:A	174427833	0	0.00E+00
NC-offspring-18	A:T→C:G	268921117	0	0.00E+00
	A:T→G:C	268921117	3	5.58E-09
	A:T→T:A	268921117	1	1.86E-09
	G:C→A:T	174788996	2	5.72E-09
	G:C→C:G	174788996	1	2.86E-09
	G:C→T:A	174788996	0	0.00E+00
	A:T→C:G	268929646	0	0.00E+00
	A:T→G:C	268929646	0	0.00E+00

Supplementary table S4 continue

NC-offspring-19	A:T→T:A	268929646	1	1.86E-09
	G:C→A:T	174794804	3	8.58E-09
	G:C→C:G	174794804	0	0.00E+00
	G:C→T:A	174794804	0	0.00E+00
NC-offspring-20	A:T→C:G	268687451	0	0.00E+00
	A:T→G:C	268687451	0	0.00E+00
	A:T→T:A	268687451	1	1.86E-09
	G:C→A:T	174641563	0	0.00E+00
	G:C→C:G	174641563	0	0.00E+00
	G:C→T:A	174641563	0	0.00E+00
NC-offspring-21	A:T→C:G	269022366	0	0.00E+00
	A:T→G:C	269022366	1	1.86E-09
	A:T→T:A	269022366	2	3.72E-09
	G:C→A:T	174854186	0	0.00E+00
	G:C→C:G	174854186	0	0.00E+00
	G:C→T:A	174854186	2	5.72E-09
NC-offspring-22	A:T→C:G	268650981	0	0.00E+00
	A:T→G:C	268650981	1	1.86E-09
	A:T→T:A	268650981	1	1.86E-09
	G:C→A:T	174620880	0	0.00E+00
	G:C→C:G	174620880	0	0.00E+00
	G:C→T:A	174620880	0	0.00E+00
NC-offspring-23	A:T→C:G	269108936	0	0.00E+00
	A:T→G:C	269108936	3	5.57E-09
	A:T→T:A	269108936	0	0.00E+00
	G:C→A:T	174909819	3	8.58E-09
	G:C→C:G	174909819	0	0.00E+00
	G:C→T:A	174909819	0	0.00E+00
NC-offspring-24	A:T→C:G	268556123	0	0.00E+00
	A:T→G:C	268556123	0	0.00E+00
	A:T→T:A	268556123	1	1.86E-09
	G:C→A:T	174557512	1	2.86E-09
	G:C→C:G	174557512	0	0.00E+00
	G:C→T:A	174557512	1	2.86E-09
NC-offspring-25	A:T→C:G	268054890	0	0.00E+00
	A:T→G:C	268054890	1	1.87E-09
	A:T→T:A	268054890	0	0.00E+00
	G:C→A:T	174247081	0	0.00E+00
	G:C→C:G	174247081	0	0.00E+00
	G:C→T:A	174247081	0	0.00E+00
NC-offspring-26	A:T→C:G	268933941	0	0.00E+00
	A:T→G:C	268933941	0	0.00E+00
	A:T→T:A	268933941	1	1.86E-09
	G:C→A:T	174798600	1	2.86E-09
	G:C→C:G	174798600	0	0.00E+00
	G:C→T:A	174798600	0	0.00E+00
NC-offspring-27	A:T→C:G	268994300	1	1.86E-09
	A:T→G:C	268994300	2	3.72E-09
	A:T→T:A	268994300	0	0.00E+00
	G:C→A:T	174835925	1	2.86E-09
	G:C→C:G	174835925	0	0.00E+00
	G:C→T:A	174835925	0	0.00E+00
NC-offspring-28	A:T→C:G	269050711	0	0.00E+00
	A:T→G:C	269050711	1	1.86E-09
	A:T→T:A	269050711	1	1.86E-09
	G:C→A:T	174868715	0	0.00E+00
	G:C→C:G	174868715	0	0.00E+00
	G:C→T:A	174868715	1	2.86E-09

NC-offspring-29	A:T→C:G	268513143	0	0.00E+00
	A:T→G:C	268513143	1	1.86E-09
	A:T→T:A	268513143	0	0.00E+00
	G:C→A:T	174528449	1	2.86E-09
	G:C→C:G	174528449	0	0.00E+00
	G:C→T:A	174528449	1	2.86E-09
NC-offspring-30	A:T→C:G	269015363	0	0.00E+00
	A:T→G:C	269015363	0	0.00E+00
	A:T→T:A	269015363	0	0.00E+00
	G:C→A:T	174848604	1	2.86E-09
	G:C→C:G	174848604	2	5.72E-09
	G:C→T:A	174848604	0	0.00E+00

**High temperature**				
**Sample**	**Mutation types**	**Callable sites**	**DNMs**	**Mutation rate**
HT-offspring-1	A:T→C:G	265048089	0	0.00E+00
	A:T→G:C	265048089	1	1.89E-09
	A:T→T:A	265048089	0	0.00E+00
	G:C→A:T	172554609	1	2.90E-09
	G:C→C:G	172554609	1	2.90E-09
	G:C→T:A	172554609	0	0.00E+00
HT-offspring-2	A:T→C:G	261746005	0	0.00E+00
	A:T→G:C	261746005	0	0.00E+00
	A:T→T:A	261746005	1	1.91E-09
	G:C→A:T	170466498	3	8.80E-09
	G:C→C:G	170466498	0	0.00E+00
	G:C→T:A	170466498	0	0.00E+00
HT-offspring-3	A:T→C:G	261772711	0	0.00E+00
	A:T→G:C	261772711	3	5.73E-09
	A:T→T:A	261772711	1	1.91E-09
	G:C→A:T	170486422	3	8.80E-09
	G:C→C:G	170486422	0	0.00E+00
	G:C→T:A	170486422	2	5.87E-09
HT-offspring-4	A:T→C:G	263335186	1	1.90E-09
	A:T→G:C	263335186	1	1.90E-09
	A:T→T:A	263335186	0	0.00E+00
	G:C→A:T	171469721	1	2.92E-09
	G:C→C:G	171469721	1	2.92E-09
	G:C→T:A	171469721	1	2.92E-09
HT-offspring-5	A:T→C:G	252798370	1	1.98E-09
	A:T→G:C	252798370	1	1.98E-09
	A:T→T:A	252798370	0	0.00E+00
	G:C→A:T	164779421	1	3.03E-09
	G:C→C:G	164779421	0	0.00E+00
	G:C→T:A	164779421	0	0.00E+00
HT-offspring-6	A:T→C:G	267105816	2	3.74E-09
	A:T→G:C	267105816	1	1.87E-09
	A:T→T:A	267105816	0	0.00E+00
	G:C→A:T	173746991	0	0.00E+00
	G:C→C:G	173746991	1	2.88E-09
	G:C→T:A	173746991	0	0.00E+00
HT-offspring-7	A:T→C:G	265101785	0	0.00E+00
	A:T→G:C	265101785	1	1.89E-09
	A:T→T:A	265101785	1	1.89E-09
	G:C→A:T	172443584	2	5.80E-09
	G:C→C:G	172443584	0	0.00E+00
	G:C→T:A	172443584	0	0.00E+00
	A:T→C:G	267483994	0	0.00E+00

HT-offspring-8	A:T→G:C	267483994	2	3.74E-09
	A:T→T:A	267483994	0	0.00E+00
	G:C→A:T	173971981	5	1.44E-08
	G:C→C:G	173971981	0	0.00E+00
	G:C→T:A	173971981	1	2.87E-09
HT-offspring-9	A:T→C:G	267169550	0	0.00E+00
	A:T→G:C	267169550	1	1.87E-09
	A:T→T:A	267169550	0	0.00E+00
	G:C→A:T	173811460	1	2.88E-09
	G:C→C:G	173811460	0	0.00E+00
	G:C→T:A	173811460	0	0.00E+00
HT-offspring-10	A:T→C:G	265606109	0	0.00E+00
	A:T→G:C	265606109	1	1.88E-09
	A:T→T:A	265606109	1	1.88E-09
	G:C→A:T	172811499	0	0.00E+00
	G:C→C:G	172811499	2	5.79E-09
	G:C→T:A	172811499	2	5.79E-09

Low temperature

Samples	Mutation types	Callable sites	DNMs	Mutation rate
LT-offspring-1	A:T→C:G	265699268	1	1.88E-09
	A:T→G:C	265699268	0	0.00E+00
	A:T→T:A	265699268	2	3.76E-09
	G:C→A:T	172958036	2	5.78E-09
	G:C→C:G	172958036	0	0.00E+00
	G:C→T:A	172958036	0	0.00E+00
LT-offspring-2	A:T→C:G	262779672	0	0.00E+00
	A:T→G:C	262779672	1	1.90E-09
	A:T→T:A	262779672	0	0.00E+00
	G:C→A:T	171127905	1	2.92E-09
	G:C→C:G	171127905	0	0.00E+00
	G:C→T:A	171127905	2	5.84E-09
LT-offspring-3	A:T→C:G	257273764	0	0.00E+00
	A:T→G:C	257273764	0	0.00E+00
	A:T→T:A	257273764	0	0.00E+00
	G:C→A:T	167625386	0	0.00E+00
	G:C→C:G	167625386	0	0.00E+00
	G:C→T:A	167625386	2	5.97E-09
LT-offspring-4	A:T→C:G	263784094	0	0.00E+00
	A:T→G:C	263784094	1	1.90E-09
	A:T→T:A	263784094	0	0.00E+00
	G:C→A:T	171776500	0	0.00E+00
	G:C→C:G	171776500	0	0.00E+00
	G:C→T:A	171776500	2	5.82E-09
LT-offspring-5	A:T→C:G	267046686	1	1.87E-09
	A:T→G:C	267046686	1	1.87E-09
	A:T→T:A	267046686	1	1.87E-09
	G:C→A:T	173815896	1	2.88E-09
	G:C→C:G	173815896	0	0.00E+00
	G:C→T:A	173815896	0	0.00E+00
LT-offspring-6	A:T→C:G	265184276	0	0.00E+00
	A:T→G:C	265184276	2	3.77E-09
	A:T→T:A	265184276	2	3.77E-09
	G:C→A:T	172440811	4	1.16E-08
	G:C→C:G	172440811	0	0.00E+00
	G:C→T:A	172440811	1	2.90E-09
	A:T→C:G	266902471	0	0.00E+00
	A:T→G:C	266902471	2	3.75E-09

Supplementary table S4 continue

LT-offspring-7	A:T→T:A	266902471	1	1.87E-09
	G:C→A:T	173667506	3	8.64E-09
	G:C→C:G	173667506	0	0.00E+00
	G:C→T:A	173667506	0	0.00E+00
LT-offspring-8	A:T→C:G	264431329	0	0.00E+00
	A:T→G:C	264431329	0	0.00E+00
	A:T→T:A	264431329	0	0.00E+00
	G:C→A:T	172033565	4	1.16E-08
	G:C→C:G	172033565	1	2.91E-09
	G:C→T:A	172033565	3	8.72E-09
LT-offspring-9	A:T→C:G	267312758	0	0.00E+00
	A:T→G:C	267312758	3	5.61E-09
	A:T→T:A	267312758	0	0.00E+00
	G:C→A:T	173883212	0	0.00E+00
	G:C→C:G	173883212	0	0.00E+00
	G:C→T:A	173883212	0	0.00E+00
LT-offspring-10	A:T→C:G	265638240	1	1.88E-09
	A:T→G:C	265638240	1	1.88E-09
	A:T→T:A	265638240	0	0.00E+00
	G:C→A:T	172788578	1	2.89E-09
	G:C→C:G	172788578	0	0.00E+00
	G:C→T:A	172788578	0	0.00E+00

Supplementary table S5. The callable sites, *de novo* mutations and mutation rate of each genome in the silkworms under different conditions.

Normal conditions				
**Samples**	**Callable sites**	**DNMs**	**Mutation rate**	**corrected mutation rate**
NC-offspring-1	443748566	5	5.63E-09	5.78E-09
NC-offspring-2	444163970	5	5.63E-09	5.77E-09
NC-offspring-3	443783376	2	2.25E-09	2.31E-09
NC-offspring-4	443808274	6	6.76E-09	6.94E-09
NC-offspring-5	443963800	0	0.00E+00	0.00E+00
NC-offspring-6	444012452	2	2.25E-09	2.31E-09
NC-offspring-7	443875003	1	1.13E-09	1.16E-09
NC-offspring-8	443181522	3	3.38E-09	3.47E-09
NC-offspring-9	444031968	3	3.38E-09	3.47E-09
NC-offspring-10	443443124	4	4.51E-09	4.63E-09
NC-offspring-11	444061936	5	5.63E-09	5.78E-09
NC-offspring-12	443829252	4	4.51E-09	4.62E-09
NC-offspring-13	442812216	8	9.03E-09	9.27E-09
NC-offspring-14	444066519	4	4.50E-09	4.62E-09
NC-offspring-15	443785597	4	4.51E-09	4.62E-09
NC-offspring-16	443434797	3	3.38E-09	3.47E-09
NC-offspring-17	442773346	3	3.39E-09	3.48E-09
NC-offspring-18	443710113	7	7.89E-09	8.09E-09
NC-offspring-19	443724450	4	4.51E-09	4.62E-09
NC-offspring-20	443329014	1	1.13E-09	1.16E-09
NC-offspring-21	443876552	5	5.63E-09	5.78E-09
NC-offspring-22	443271861	2	2.26E-09	2.31E-09
NC-offspring-23	444018755	6	6.76E-09	6.93E-09
NC-offspring-24	443113635	3	3.39E-09	3.47E-09
NC-offspring-25	442301971	1	1.13E-09	1.16E-09
NC-offspring-26	443732541	2	2.25E-09	2.31E-09
NC-offspring-27	443830225	4	4.51E-09	4.62E-09
NC-offspring-28	443919426	3	3.38E-09	3.47E-09
NC-offspring-29	443041592	3	3.39E-09	3.47E-09
NC-offspring-30	443863967	3	3.38E-09	3.47E-09

Low temperature				
**Samples**	**Callable sites**	**DNMs**	**Mutation rate**	**corrected mutation rate**
LT-offspring-1	438657304	5	5.70E-09	6.02E-09
LT-offspring-2	433907577	4	4.61E-09	4.87E-09
LT-offspring-3	424899150	2	2.35E-09	2.49E-09
LT-offspring-4	435560594	3	3.44E-09	3.64E-09
LT-offspring-5	440862582	4	4.54E-09	4.79E-09
LT-offspring-6	437625087	9	1.03E-08	1.09E-08
LT-offspring-7	440569977	6	6.81E-09	7.19E-09
LT-offspring-8	436464894	8	9.16E-09	9.68E-09
LT-offspring-9	441195970	3	3.40E-09	3.59E-09
LT-offspring-10	438426818	3	3.42E-09	3.61E-09

Supplementary table S5 continue

High temperature				
**Samples**	**Callable sites**	**DNMs**	**Mutation rate**	**corrected mutation rate**
HT-offspring-1	437602698	3	3.43E-09	3.61E-09
HT-offspring-2	432212503	4	4.63E-09	4.87E-09
HT-offspring-3	432259133	9	1.04E-08	1.10E-08
HT-offspring-4	434804907	5	5.75E-09	6.05E-09
HT-offspring-5	417577791	3	3.59E-09	3.78E-09
HT-offspring-6	440852807	4	4.54E-09	4.78E-09
HT-offspring-7	437545369	4	4.57E-09	4.81E-09
HT-offspring-8	441455975	8	9.06E-09	9.54E-09
HT-offspring-9	440981010	2	2.27E-09	2.39E-09
HT-offspring-10	438417608	6	6.84E-09	7.21E-09

Supplementary table S6. The callable sites, *de novo* mutations and mutation rate in gene region and intergenic regions of each genome.

Normal condition								
Samples	Gene region				Intergenic region			
	DNMs	Callable sites	mutation rate	Corrected mutation rate	DNMs	Callable sites	mutation rate	Corrected mutation rate
NC-offspring-1	2	159045881	6.29E-09	6.45E-09	3	284702685	5.27E-09	5.41E-09
NC-offspring-2	0	159132594	0.00E+00	0.00E+00	5	285031376	8.77E-09	9.00E-09
NC-offspring-3	2	159048414	6.29E-09	6.45E-09	0	284734962	0.00E+00	0.00E+00
NC-offspring-4	0	159046059	0.00E+00	0.00E+00	6	284762215	1.05E-08	1.08E-08
NC-offspring-5	0	159078554	0.00E+00	0.00E+00	0	284885246	0.00E+00	0.00E+00
NC-offspring-6	1	159105079	3.14E-09	3.22E-09	1	284907373	1.75E-09	1.80E-09
NC-offspring-7	0	159079636	0.00E+00	0.00E+00	1	284795367	1.76E-09	1.80E-09
NC-offspring-8	1	158934688	3.15E-09	3.23E-09	2	284246834	3.52E-09	3.61E-09
NC-offspring-9	0	159114353	0.00E+00	0.00E+00	3	284917615	5.26E-09	5.40E-09
NC-offspring-10	2	158989674	6.29E-09	6.45E-09	2	284453450	3.52E-09	3.61E-09
NC-offspring-11	2	159120494	6.28E-09	6.45E-09	3	284941442	5.26E-09	5.40E-09
NC-offspring-12	1	159068964	3.14E-09	3.23E-09	3	284760288	5.27E-09	5.40E-09
NC-offspring-13	5	158821391	1.57E-08	1.62E-08	3	283990825	5.28E-09	5.42E-09
NC-offspring-14	2	159121383	6.28E-09	6.45E-09	2	284945136	3.51E-09	3.60E-09
NC-offspring-15	3	159063137	9.43E-09	9.68E-09	1	284722460	1.76E-09	1.80E-09
NC-offspring-16	1	158866158	3.15E-09	3.23E-09	2	284568639	3.51E-09	3.61E-09
NC-offspring-17	1	158643557	3.15E-09	3.23E-09	2	284129789	3.52E-09	3.61E-09
NC-offspring-18	2	158978312	6.29E-09	6.45E-09	5	284731801	8.78E-09	9.01E-09
NC-offspring-19	0	158989264	0.00E+00	0.00E+00	4	284735186	7.02E-09	7.21E-09
NC-offspring-20	0	158829931	0.00E+00	0.00E+00	1	284499083	1.76E-09	1.80E-09
NC-offspring-21	2	159033742	6.29E-09	6.45E-09	3	284842810	5.27E-09	5.40E-09
NC-offspring-22	2	158811220	6.30E-09	6.46E-09	0	284460641	0.00E+00	0.00E+00
NC-offspring-23	4	159081674	1.26E-08	1.29E-08	2	284937081	3.51E-09	3.60E-09
NC-offspring-24	1	158752340	3.15E-09	3.23E-09	2	284361295	3.52E-09	3.61E-09
NC-offspring-25	0	158436319	0.00E+00	0.00E+00	1	283865652	1.76E-09	1.81E-09
NC-offspring-26	1	158980824	3.15E-09	3.23E-09	1	284751717	1.76E-09	1.80E-09
NC-offspring-27	1	159021187	3.14E-09	3.23E-09	3	284809038	5.27E-09	5.40E-09
NC-offspring-28	2	159053369	6.29E-09	6.45E-09	1	284866057	1.76E-09	1.80E-09
NC-offspring-29	2	158735819	6.30E-09	6.46E-09	1	284305773	1.76E-09	1.80E-09
NC-offspring-30	2	159031623	6.29E-09	6.45E-09	1	284832344	1.76E-09	1.80E-09

Supplementary table S6 continue

High temperature								
Samples	Gene region				Intergenic region			
	DNMs	Callable sites	Mutation rate	Corrected mutation rate	DNMs	Callable sites	Mutation rate	Corrected mutation rate
HT-offspring-1	2	156396731	6.39E-09	6.56E-09	1	281205967	1.78E-09	1.82E-09
HT-offspring-2	0	154725845	0.00E+00	0.00E+00	4	277486658	7.21E-09	7.39E-09
HT-offspring-3	3	154612026	9.70E-09	9.95E-09	6	277647107	1.08E-08	1.11E-08
HT-offspring-4	2	155458074	6.43E-09	6.60E-09	3	279346833	5.37E-09	5.51E-09
HT-offspring-5	2	149686949	6.68E-09	6.85E-09	1	267890842	1.87E-09	1.91E-09
HT-offspring-6	2	157960066	6.33E-09	6.50E-09	2	282892741	3.53E-09	3.63E-09
HT-offspring-7	1	156968771	3.19E-09	3.27E-09	3	280576598	5.35E-09	5.49E-09
HT-offspring-8	3	158111213	9.49E-09	9.73E-09	5	283344762	8.82E-09	9.05E-09
HT-offspring-9	0	157969221	0.00E+00	0.00E+00	2	283011789	3.53E-09	3.63E-09
HT-offspring-10	0	157192671	0.00E+00	0.00E+00	6	281224937	1.07E-08	1.09E-08

Low temperature								
Samples	Gene region				Intergenic region			
	DNMs	Callable sites	Mutation rate	Corrected mutation rate	DNMs	Callable sites	Mutation rate	Corrected mutation rate
LT-offspring-1	2	156764126	6.38E-09	6.54E-09	3	281893178	5.32E-09	5.46E-09
LT-offspring-2	2	155122214	6.45E-09	6.61E-09	2	278785363	3.59E-09	3.68E-09
LT-offspring-3	1	152302751	3.28E-09	3.37E-09	1	272596399	1.83E-09	1.88E-09
LT-offspring-4	1	155687414	3.21E-09	3.30E-09	2	279873180	3.57E-09	3.67E-09
LT-offspring-5	1	157606647	3.17E-09	3.25E-09	3	283255935	5.30E-09	5.43E-09
LT-offspring-6	2	157034368	6.37E-09	6.53E-09	7	280590719	1.25E-08	1.28E-08
LT-offspring-7	2	157898935	6.33E-09	6.50E-09	4	282671042	7.08E-09	7.26E-09
LT-offspring-8	2	156641725	6.38E-09	6.55E-09	6	279823169	1.07E-08	1.10E-08
LT-offspring-9	1	158122658	3.16E-09	3.24E-09	2	283073312	3.53E-09	3.62E-09
LT-offspring-10	1	157272803	3.18E-09	3.26E-09	2	281154015	3.56E-09	3.65E-09
