# Supplementary material for: Mutation Rate and Spectrum of the Silkworm in Normal and Temperature Stress Conditions
Source: Genes (Basel). 2023 Mar 4;14(3):649. doi: 10.3390/genes14030649 (PMC10048334; doi:10.3390/genes14030649)
Supplement: Supplementary file 1 [file genes-14-00649-s001.zip › Supplementary_Figures S1-S6.pdf]

Supplementary Figures

A

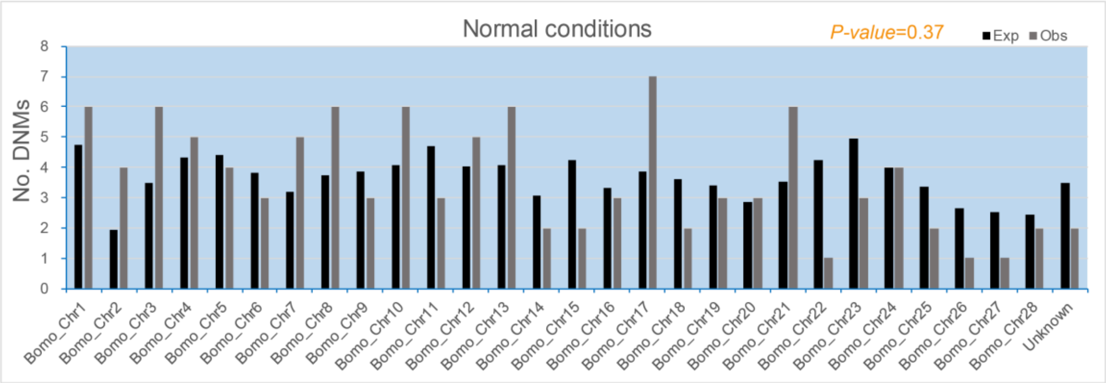

B

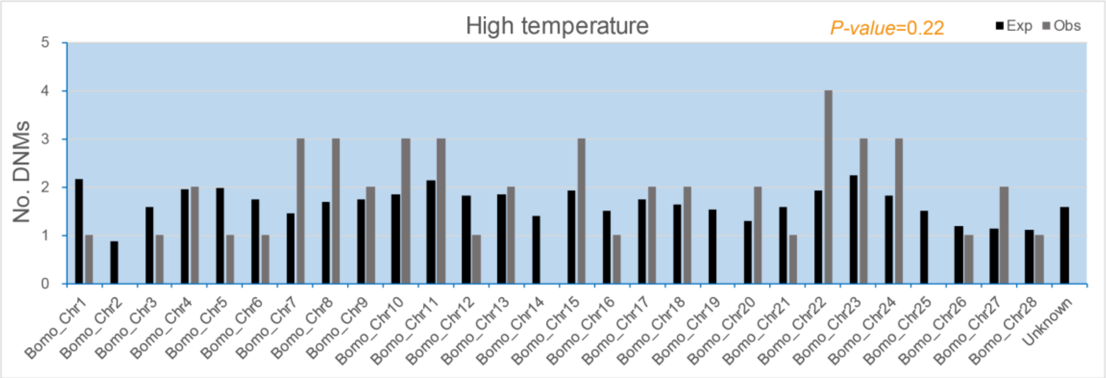

C

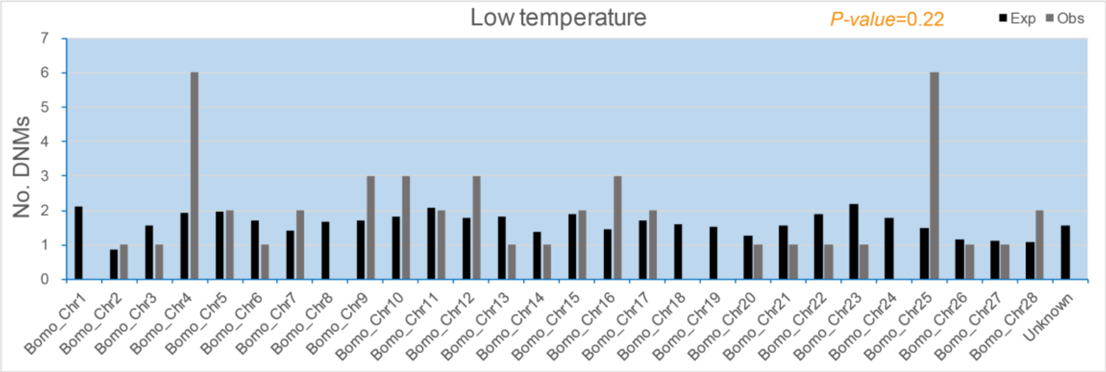

Supplementary Figure S1. The expected distributions and the observed distributions of the *de novo* mutations on each chromosome of the silkworm under different conditions. For each group, difference of the *de novo* mutation distribution between observed and expected values was assessed by a K-S test.

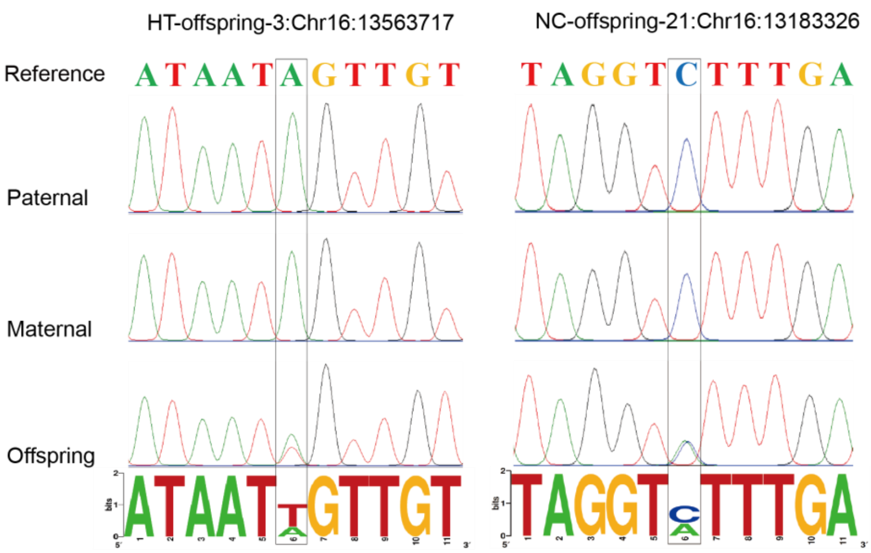

Supplementary Figure S2. Two examples of PCR and Sanger sequencing verifications. The top line and black box show the chromosome position and the corresponding point mutation site in the offspring. Images of Sanger sequencing peaks are shown in the middle regions. When the two parents show a unique peak at mutation sites, the offspring shows two peaks at the corresponding site. The sequences (from Illumina sequencing reads of mutation site) logos of the mutation region including 10 bp of the flank of mutation site are shown at the bottom.

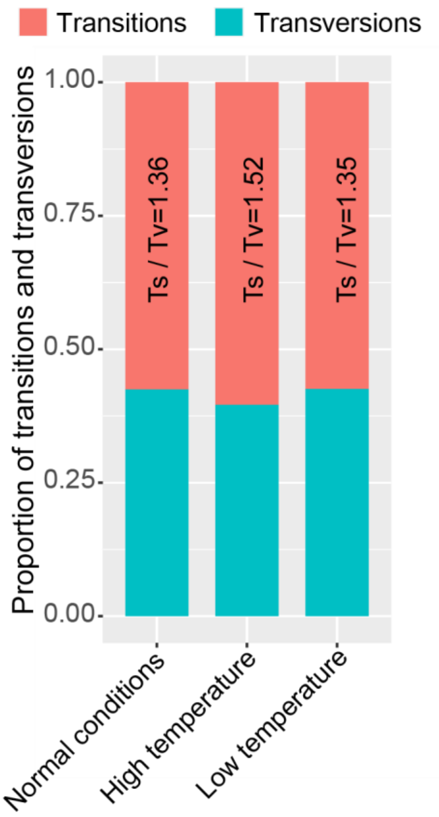

Supplementary Figure S3. The proportions of transitions and transversions as well as the ratios of transitions to transversions of the silkworm under different conditions.

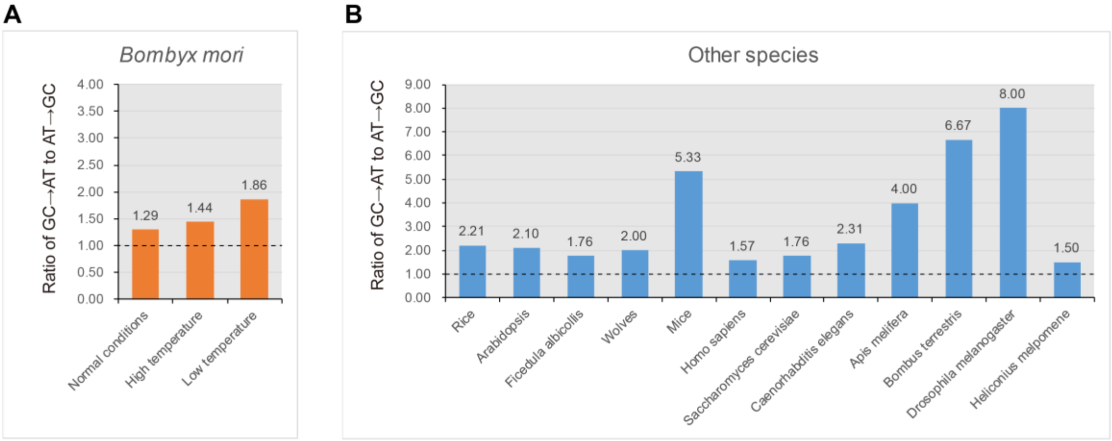

Supplementary Figure S4. The ratios of the GC→AT mutations to the AT→GC mutations of (**A**) the silkworms under different conditions and (**B**) other species. The data of other species are summarized from previous studies (References: *Heliconius melpomene* (Keightley et al. 2015); *Drosophila melanogaster* (Keightley et al. 2014); *Bombus terrestris* (Liu et al. 2017); *Apis mellifera*, rice and Arabidopsis (Yang et al. 2015); *Caenorhabditis elegans* (Denver et al. 2012); *Saccharomyces cerevisiae* (Liu and Zhang 2019); *Homo sapiens* (Rahbari et al. 2016); Mice (Uchimura et al. 2016); Wolves (Koch et al. 2019); and *Ficedula albicollis* (Smeds et al. 2016)).

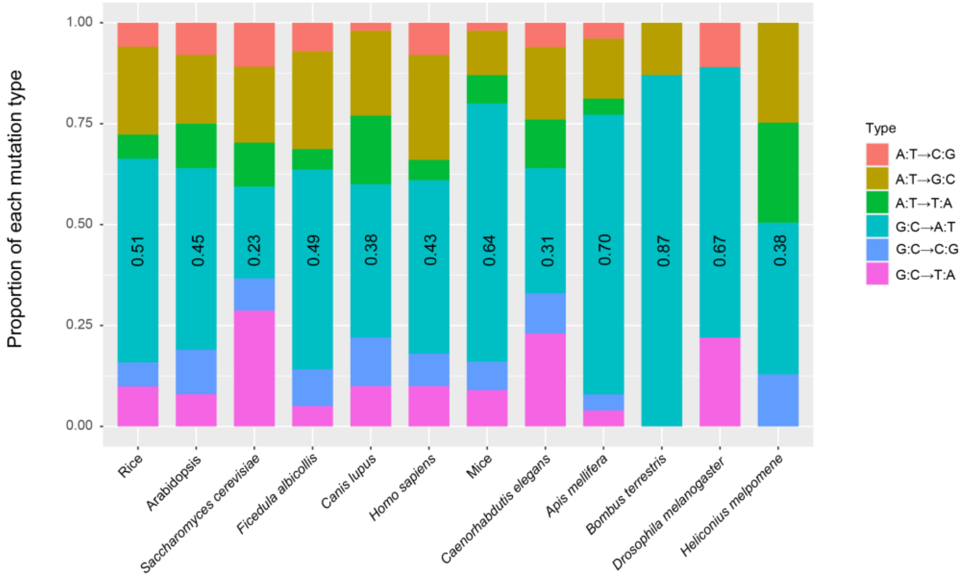

Supplementary Figure S5. The proportions of six mutation types of other species are summarized from previous studies (the references are the same as other species of supplementary Figure S4).

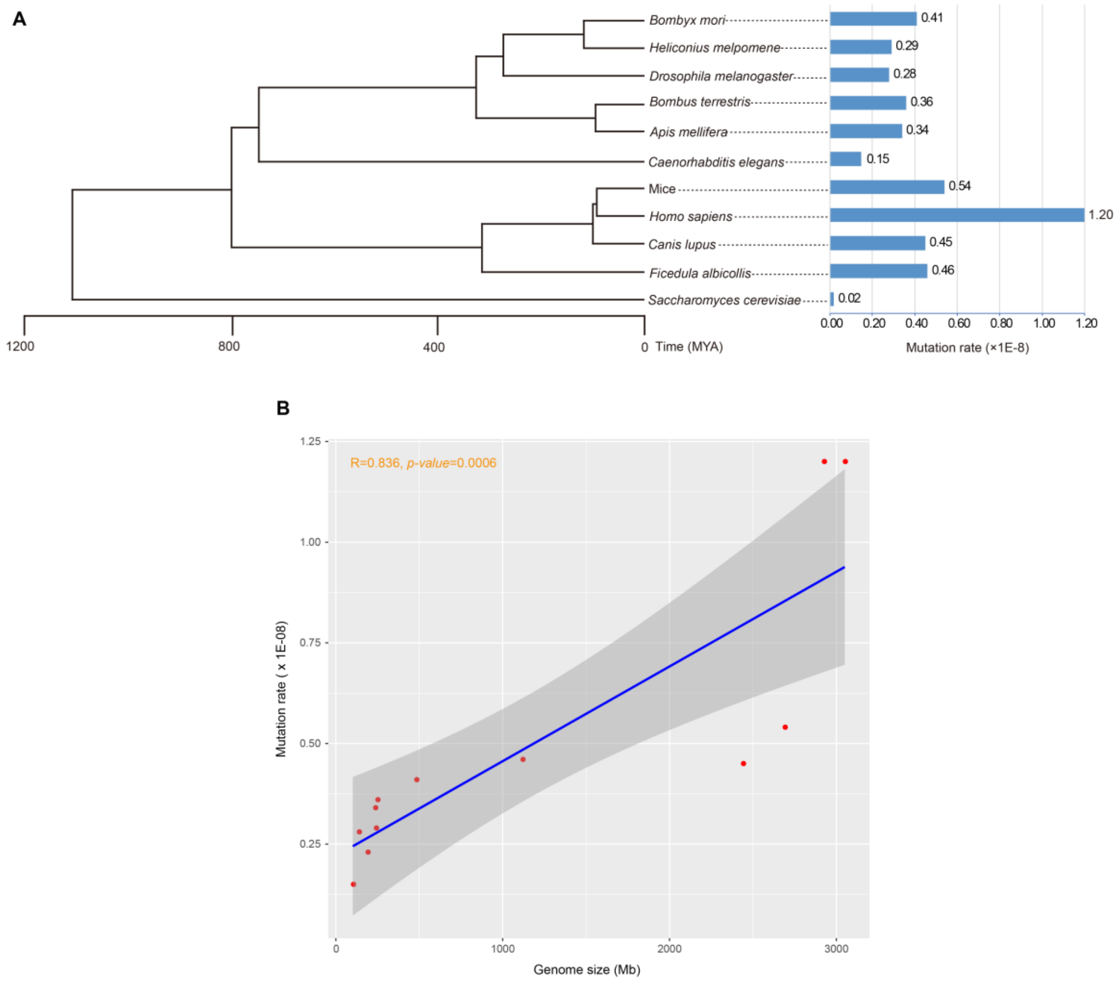

Supplementary Figure S6. (A) The mutation rate of different species. The estimate of the silkworm was obtained from this study. The estimates of other species are from previous studies (the references are the same as other species of supplementary Figure S4). The phylogenetic tree was generated by TimeTree (http://www.timetree.org/). (B) Correlation analysis between the mutation rate and genome size was performed using the R program with Pearson's method.
